# Nanotechnology Enabled Enhancement of Enzyme Activity and Thermostability: Study on Impaired Pectate Lyase from Attenuated *Macrophomina phaseolina* in Presence of Hydroxyapatite Nanoparticle

**DOI:** 10.1371/journal.pone.0063567

**Published:** 2013-05-15

**Authors:** Nalok Dutta, Arka Mukhopadhyay, Anjan Kr. Dasgupta, Krishanu Chakrabarti

**Affiliations:** Department of Biochemistry, University of Calcutta, Kolkata, India; Jacobs University Bremen, Germany

## Abstract

In this paper we show that hydroxyapatite nanoparticles (NP) can not only act as a chaperon (by imparting thermostability) but can serve as a synthetic enhancer of activity of an isolated extracellular pectate lyase (APL) with low native state activity. The purified enzyme (an attenuated strain of *Macrophomina phaseolina*) showed feeble activity at 50°C and pH 5.6. However, on addition of 10.5 µg/ml of hydroxyapatite nanoparticles (NP), APL activity increased 27.7 fold with a 51 fold increase in half-life at a temperature of 90°C as compared to untreated APL. The chaperon like activity of NP was evident from entropy–enthalpy compensation profile of APL. The upper critical temperature for such compensation was elevated from 50°C to 90°C in presence of NP. This dual role of NP in enhancing activity and conferring thermostability to a functionally impaired enzyme is reported for the first time.

## Introduction

Pectate lyases (PL) (EC 4. 2. 2. 2) are enzymes involved in soft rotting of plant tissue.

The best studied microbial PLs to date are those from *Erwinia chrysanthemi*, which causes devastating diseases involving maceration of parenchymatous tissues of various dicot plants [1], [2], [3]. These enzymes act by depolymerizing cell-wall polygalacturonides in the presence of calcium ions, thus destroying the integrity of the plant tissues. *Erwinia* isoforms, obtained by expression in *E. coli*, have been shown to act synergistically to extend the range of pectin substrates that the bacterium can degrade [4]. *Macrophomina phaseolina* has been reported to produce an endo pectin lyase in the culture filtrate during growth [5].The enzyme catalyzes the eliminative cleavage of (1->4)-alpha-D-galacturonan to give oligosaccharides with 4-deoxy-alpha-D-galact-4-enuronosyl groups at their non-reducing ends. Reports indicate that endo-polygalacturonase and endo-polygalacturonic acid lyase, are elicitors of plant disease resistance [6], [7].

Pectins, a major constituent of cereals, vegetables, fruits and fibres, are complex, high molecular weight, heterogeneous and acidic structural polysaccharides. D-Galacturonic acid is one of the major components of pectins. Additionally, rhamnose constitutes a minor component of the pectin backbone where as other neutral sugars such as arabinose, galactose and xylose are also present in the side chains. Pectinolytic enzymes that hydrolyze pectins find applications in various industrial processes. Based on pH requirement for optimum enzymatic activity, pectinases can be broadly classified into acidic and alkaline pectinases. Acidic pectinases are useful for extraction, clarification and liquefaction of fruit juices [8] and wines [9]. Alkaline pectinases are widely used in the fabric industry, for retting of plant fibres such as flax, hemp and jute, bio-preparation of cotton fabrics, enzymatic polishing of jute and cotton blended fabrics, in the paper industry, in the treatment of pulp and paper mill effluents and for improving the quality of black tea. Pectinases account for a 25% share of the global sales of food enzymes [9], [10], [11].

Scouring of natural fibers by pectinase enzyme improves water absorbency and whiteness of textiles by removing non-cellulosic substances from many natural fibers [12]. Chemicals like soda-ash, oxalic acid, caustic soda, used in chemical scouring process give rise to polluting effluents accompanied with weakening of the strength of the processed fiber. Using pectinase thus gives an option of a bio friendly process. Moreover, as scouring requires high temperatures, thermostability of the used enzyme comes into contention.

Nanostructure materials exhibit interesting properties such as a large surface-to-volume ratio, high surface reaction activity, high catalytic efficiency, and strong adsorption ability. Adsorption leads to improved performance in terms of activity [13]. The use of nanoparticles for enzyme supports was first reported in the late 1980s [14], [15]. Significant improvement in thermal stability of a lipase from *Candida rugosa* immobilized on polylactic acid nanoparticles was observed after adsorption [16]. Stability of keratin enzyme was maximized by the use of nanoscaled support [17]. The large surface area of nanomaterials possibly provides for a better matrix for the immobilization of enzymes, leading to increased enzyme loading per unit mass of particles. The multipoint attachment of enzyme molecules to nanomaterial surfaces reduces protein unfolding; resulting in the enhanced stability of the enzyme attached to the nanoparticle surfaces. The enzyme-attached nanoparticles facilitate enzymes to act as free enzymes in solution and in turn improve the enzyme-substrate interaction by avoiding the potential aggregation of the free enzyme. Some recent reports show that nanoparticles can also act as chaperon and that assists the native structures of proteins to prevail [18].

We have recently reported that metal co-factors (Ca and Cu) for bacterial pectate lyase and laccase, respectively, when added as nano-particles conferred thermostability and helped retention of activity of the enzyme. In this study we have examined the effect of NP (calcium hydroxyapatite nano-particle) on the activity of pectate lyase (PL), obtained from *Macrophomina phaseolina*. In addition to the activity retention and thermostability enhancement as we have reported earlier [19] in the presence of NP we observed an elevation of native state activity of this purified enzyme. Incidentally this PL is isolated from an attenuated organism, which is functionally impaired. Both activity measurements *vis a vis* thermodynamic studies validated our basic observations. On the broader application side, the findings provide a means of using a non-virulent fungus as a source of pectate lyase. From the nanotechnology perspective the dual role of nano-particles in restoration of activity of a functionally impaired enzyme and acting as a chaperon by imparting additional thermostability, pose a new nano-biotechnology perspective.

## Materials and Methods

The NP nanoparticle dispersion, 10 wt, % in H_2_O <200 nm (Acc No: 702153) was supplied by SIGMA-ALDRICH. All other chemicals were also obtained from SIGMA-ALDRICH and were of analytical grade.

### Preparation of Fungal Culture

A virulent isolate of *Macrophomina phaseolina* (strain R9) was collected from Central Research Institute for jute and Allied fibres (CRIJAF), Barrackpore, West Bengal. A pure mycelial culture generated through single spore of this isolate, maintained in Potato Dextrose Agar (PDA) media at 30°C, served as the initial source of inoculum. For the mass culture, the pathogen was grown in Potato Dextrose Broth (PDB) and incubated at 30°C for 72 h. This strain viz. the wild type fungal strain is referred to as WT.

### Preparation of the Attenuated Fungal Strain

The virulent fungus, grown on Potato Dextrose Agar (PDA), was kept under UV illumination of Klenz Flow-Laminar Air Flow work station for upto 60 hours. The treated cultures were re-cultured in PDB for five generations. The virulence of the fungal strain was monitored by inoculating intact detached leaves of sunflower, kept on moist filter paper with the UV exposed fungus taken from a mycelial bed of Macrophomina (strain R9), from the 30^th^ and the 60^th^ hour of UV irradiation (using a sterile tooth pick). The fungal culture (60 hours post irradiation) lost its virulence as evident from lack of lesions (as compared to leaves inoculated with sterile PDB media). This attenuated strain is referred to as APL. (The re-cultured APL retained its attenuation in all subsequent cultures.).

### Comparison of Gene Sequences of Pectate Lyase Obtained from APL and WT

We isolated the genomic DNA from both the WT and the APL by the method based on [20]. Partial amplification of the 16S rRNA gene was performed with the thermal cycler ABI 9700 (ABI, Foster City, USA). The amplified and gel-eluted PCR fragments were sequenced with an ABI 3100 Genetic Analyzer. The blastn software of the NCBI GenBank confirmed the identity of *Macrophomina phaseolina* for both WT and APL. Primers specific for pectate lyase were designed for WT and APL from consensus sequences of *Macrophomina phaseolina.* The specific PCR products were eluted from agarose gel using Qiagen gel elution kit and purified. The purified gel-eluted PCR fragments were sequenced in ABI 3100 Genetic Analyzer. The nucleotide sequences were deposited in the EMBL nucleotide sequence database under the accession number HF565055 for the WT and HF565056 for the APL.

### Purification of the Pectate Lyase from WT and APL

Pectate lyase was purified from 100 ml PDB culture broth containing 0.75% apple pectin. After 48 hours culture, the culture filtrate (100 ml) was precipitated with 80% saturation of ammonium sulphate followed by dialysis.

All steps of the purification procedure were performed at 4°C. The dialysed protein was loaded onto a DEAE-Sepharose column that had been pre-equilibrated with 20 mM acetate buffer (pH 5.6) and allowed to equilibrate for 12 hours. After washing the column with the acetate buffer, a 60 ml increasing discontinuous gradient (0–200 mM) of NaCl dissolved in 20 mM acetate buffer (pH 5.6) was applied to the column. Proteins were eluted in fractions of 1 ml. The fractions showing pectate lyase activity (assayed as below) were concentrated using a Macrosep 10 K unit and loaded onto a glass column packed with Sephadex G-75 (bed volume 30 ml) and equilibrated with the acetate buffer. Elution of the proteins was done using the same buffer. The collection of the fractions and assay enzyme activity were as described below. Fractions were run on 12% SDS polyacrylamide according to [21] using Bio-Rad electrophoresis apparatus. Amount of protein that was loaded on SDS gel page lanes was 0.38 mg/ml for both the WT-PL and the APL. Protein markers and the protein bands were stained by silver staining [22].

### Activity Assay of Pectate Lyase

The purified pectate lyase activity was measured by TBA (thio-barbituric acid) method [14]. The pectate lyase fractions were incubated with 0.015% poly-galactouronic acid (PGA) in the presence hydroxyapatite nanoparticle (NP-WT or NP-APL) or in presence of 1 mM CaCl_2_ (WT or APL) or in the absence of either WT (C) or APL (C) in 20 mM acetate buffer (pH 5.6) for 2 hours at 50°C. The assay volume was made upto 1 ml including the buffer, substrate and enzyme. After incubation, 9% (w/v) zinc sulphate and 0.5 M sodium hydroxide were added to stop the reaction. The sample was centrifuged at 3000 g for 10 minutes at 4°C. The clear supernatant was mixed with 0.04 M TBA and 0.1 M HCl followed by 30 minutes heating in boiling water. The colour formation was detected at 550 nm. One unit of activity was defined as the amount of enzyme that caused a change in the absorbance of 0.01 under the conditions of the assay.

### Measurement of Ca Concentration by Atomic Absorption Spectra (AAS)

The Ca content of NP and 1 mM CaCl_2_, was measured using AAS technique (Analyst 200, Perkin Elmer). Standard Ca ion solution was provided by Perkin Elmer. NP of different dilutions (0.005 M, 0.01 M, 0.015 M, 0.02 M,0.025 M, 0.03 M and 0.035 M) were prepared and their concentration was determined by comparison of data with the standard solutions provided by Perkin Elmer.

### Effect of Nano-particles on Pectate Lyase Activity

NP at final concentrations of 2.1 µg/ml, 4.2 µg/ml, 6.3 µg/ml, 8.4 µg/ml, 10.5 µg/ml, 12.6 µg/ml and 14.7 µg/ml were incubated with purified PL (200 µl of 0.145 mg/ml) at 50–90°C and the enzyme activity of each system was measured as described above.

### Effect of Temperature on NP-WT and NP-APL

The optimum temperature for activity of NP-WT and NP-APL was determined by carrying out the standard assay in acetate buffer (20 mM, pH 5.6), at temperatures ranging from 50° to 90°C. At each temperature the assay mixture was incubated for 2 hours. WT and APL (with 1 mM CaCl_2_ served as controls in this and all subsequent experiments).

### Thermal Stability of NP-WT and NP-APL

To measure the retention of enzyme activity (with PGA as substrate) at high temperature, NP-WT and NP-APL were incubated for 1–6 hours at 90°C. After the treatment, the enzyme activities were determined as above. The results were expressed relative to the values of NP treated similarly. The experiments were repeated using 0.015% apple pectin (Sigma-Aldrich) as substrate.

### Kinetics and Activation-inactivation Parameters NP-WT and NP-APL

The Km-Vmax, activation energy (E_a_) and the activation/deactivation kinetics were studied using the standard reaction mix as described above. For the study of enzyme kinetics of pectate lyase, the buffer (20 mM acetate buffer, pH 5.6) contained 10.5 µg/ml NP. The enzyme concentration was 0.15 mg/ml for both the systems.

### Km, Vmax and Activation Energy (E_a_)

The kinetic parameters, Km, V_max_ and the activation energy (E_a_) were measured according to [23].The substrate (PGA) concentration used was from 0.015% to 1.25% to determine K_m_ and V_max_. Since the optimum temperature required for the purified WT and APL was 50°C, we carried out one set of experiments for K_m_, V_max_ at this temperature. Since high activities for NP-WT and NP-APL were obtained at 90°C a separate set of experiment was performed at this temperature. The activation energy (E_a_) was evaluated for the temperature range of 50° –90°C. The PGA concentration used for this calculation was 0.75% [12].

### Activation/inactivation Kinetics of NP-WT and NP-APL

The systems were pre-treated to temperatures between 40°C and 90°C (313–363 K) for up to 10 min. Inactivation parameters comprising half-life (t_1/2_), decay rate constant (k), energy of deactivation (E_d_), enthalpy (ΔH), entropy (ΔS) and free energy change (ΔG) were obtained according to [24]. The PGA concentration used for this purpose was 0.75% [24].

### CD Spectroscopy

CD spectra over the range of 190–250 nm were obtained for the systems, WT (+ CaCl_2_), APL (+ CaCl_2_), NP-WT, NP-APL using an Applied Photosystem Chirascan spectropolarimeter. Enzyme concentration was maintained at 0.38 mg/ml for all the observations. The spectral analyses were carried out with 1 mm pathlength cell and 1 nm bandwidth for every observation. Collections were done with a scan rate of 20 nm/min. For the spectral measurements, a system of (20 mM acetate buffer, pH 5.6) was used as blank.

## Results

### Analysis of the Sequencing Data

**Figure 1A:**
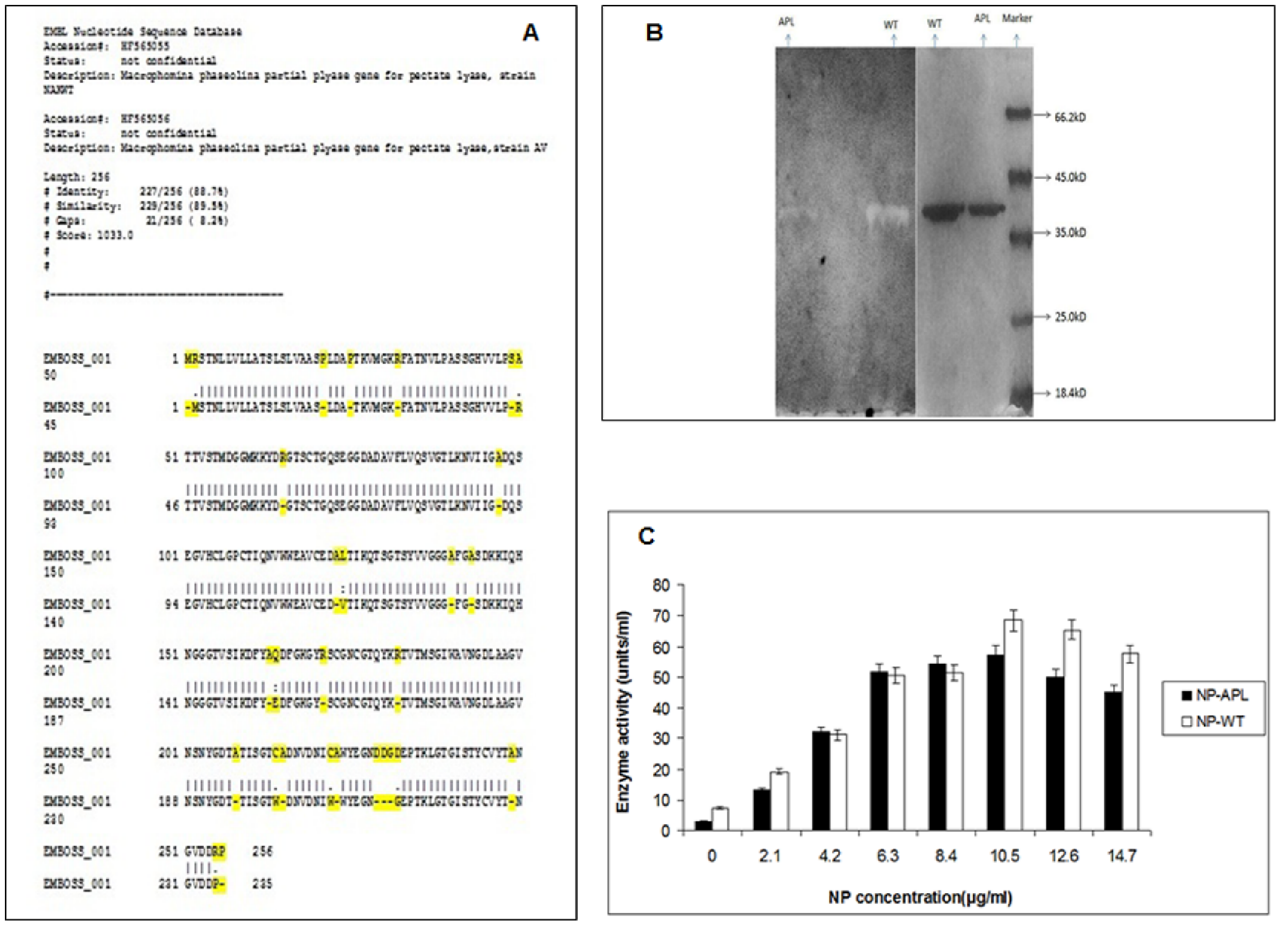
Alignment of the protein sequences of WT and APL using EMBOSS Needle: Pairwise Sequence Alignment (PROTEIN). Figure 1B. Purified APL and the WT on 12% SDS-PAGE gel (right) and ruthenium red stained zymogram gel containing 0.1% PGA (left) showing purified APL and WT. Figure 1C. NP concentration required for enzymatic activity.

The nucleotide sequences were checked by the blastp software of NCBI GenBank which confirmed the pectate lyase sequences. Thereafter, the protein sequences of the WT and the APL were aligned using **EMBOSS Needle | Pairwise Sequence Alignment | EBI (PROTEIN)** (http://www.ebi.ac.uk/Tools/psa/emboss_needle/) Accessed 2012 Nov. Several amino acid deletions and substitutions were found for the APL compared to the WT.(Fig: 1A).

Deletions were encountered for amino acids namely alanine (A),Arginine (R), Proline (P),Aspartic acid (D) and Serine (S) at several positions in the APL sequence as compared to the WT sequence.

Substitutions of amino acids in the form Arginine (R), Cysteine (C), Leucine (L),Glutamine (Q) at various positions in the APL sequence as compared to the WT sequence.

### Purification of WT and APL

The purified WT and APL (Fig: 1B) did not show any difference in the molecular weights. However, the APL had only about 27% activity as compared to WT [Table 1 and Table 2]. Protein molecular weight marker of Fermentas life science was used. (#SM0431, Lot 2411). The identification of the purified WT and the APL was done by zymogram staining after SDS gel electrophoresis [25]. The zymogram showed a single band for the purified protein thereby confirming that the purified protein and enzyme were identical.

**Table 1:** Purification of Pectate lyase from attenuated strain of *Macrophomina phaseolina*.

Purification Steps	Total Volume (ml)	Total Protein (mg)	Total Activity (Unit)	Specific Activity (Unit/mg)	Purification (fold)	Yield (%)
**Crude**	100	88	1335	15.1	1.0	100
**(080)% (NH4)_2_SO_4_Saturation**	15	21.3	845	39.7	2.6	63.3
**DEAE-Sepharose**	10	8.4	451	53.7	3.5	33.8
**Gel filtration (Sephadex G-75)**	5	3.2	203	63.4	4.2	15.2

**Table 2:** Purification of Pectate lyase from the WT strain of *Macrophomina phaseolina*.

Purification Steps	Total Volume(ml)	Total Protein (mg)	Total Activity (Unit)	Specific Activity (Unit/mg)	Purification (fold)	Yield (%)
**Crude**	100	93	5327	57.2	1.0	100
**(0–80)% (NH4)_2_SO_4_ Saturation**	15	32.5	3687	113.4	1.9	69.2
**DEAE-Sepharose**	10	14	2590	185	3.2	48.6
**Gel filtration (Sephadex G-75)**	5	8	1860	232.5	4.0	34.9

### Ca content in NP

From the AAS absorbance studies, it was found that 1 mM CaCl_2_ contain 250% higher Ca^2+^ than NP when considered on a per µl basis. (Table 3).

**Table 3:** Calcium concentration of hydroxyapatite nanoparticles by Atomic Absorption Spectra.

System	1 mM CaCl_2_	Hydroxyapatite NP
**Ca^2+^ concentration** **(µg/µl)**	1.47±0.017	0.42±0.014

### Effect of NP Concentrations on Activities of WT and APL at 50°C

**Figure 2:**
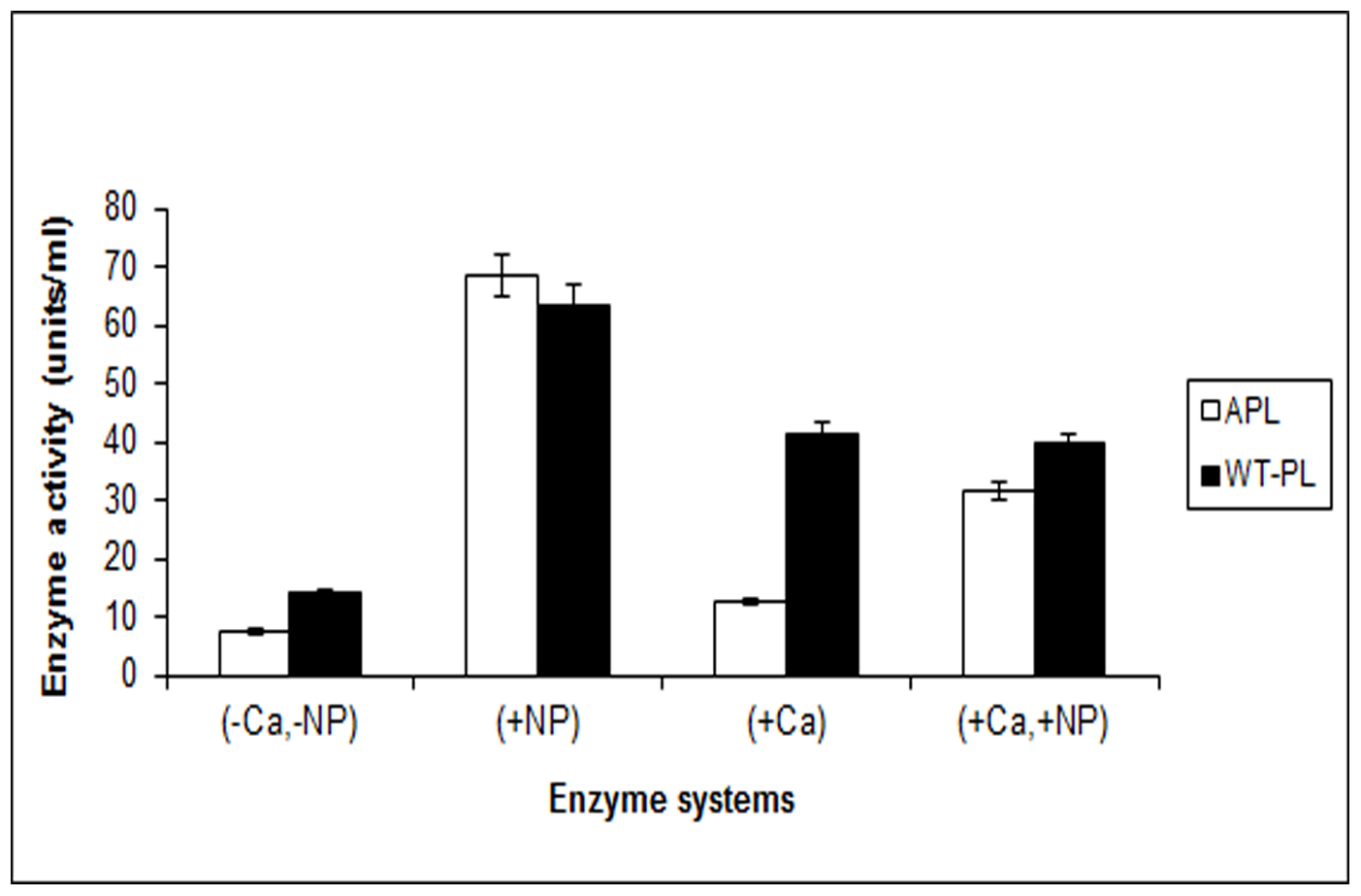
NP affecting PL activity at 50°C.

Activity of the purified APL increased with increasing concentrations of NP reaching a maximum at 10.5 µg/ml concentration. This corresponded to an increase of about 19 fold activity as compared to untreated APL. For WT the trend was similar to APL. The increase for WT was about 9 fold as compared to untreated WT. The activity of NP-APL and NP-WT were similar over the concentration range of 4.2–8.45 µg/ml. Thereafter, NP-WT was slightly higher than NP-APL with a gradually decreasing trend. The decrease (from optimum levels) was 16.1% for NP-WT and 21.2% for the NP-APL (Fig: 1C). These results are remarkable when compared to the activities of APL (12.4 Units/ml) and WT (41.5 Units/ml) in the presence of 1 mM CaCl_2_ (which was the optimum concentration for enzyme activity as determined in our earlier experiments; data not shown). As evident (from Fig: 1C and 2) 1 mM CaCl_2_ could not restore activity to APL which remained at about 50% of WT levels. A further significant observation was the inability of NP to restore the activity of APL in the presence of 1 mM CaCl_2_. This is in keeping with the findings of [19] with purified pectate lyase from *Bacillus pumilus*. Thus, NP provided higher activation/restoration of WT/APL at 10.5 µg in the system as compared to 73.5 µg Ca supplied by 1 mM CaCl_2_.

### Effect of Temperature on NP-WT and NP-APL (PGA as Substrate)

**Figure 3A:**
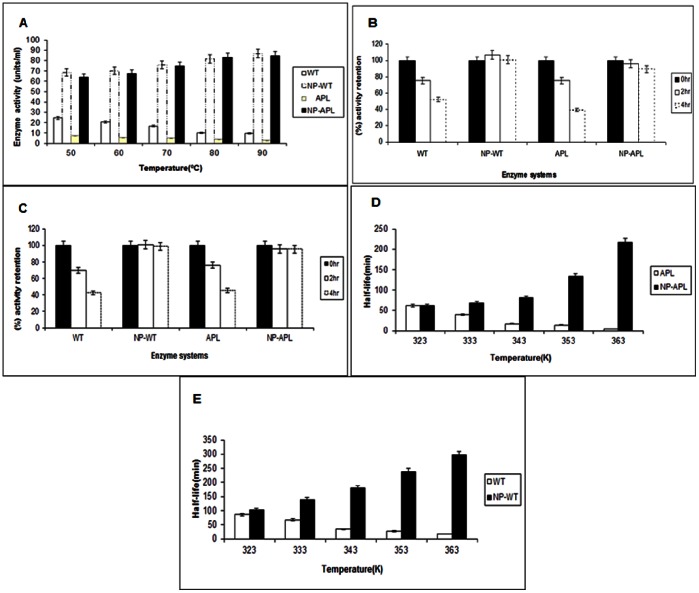
Variation of PL activity as a function of temperature. Figure 3B. Retention of PL activity when Pectin is the substrate. Figure 3C. Retention of PL activity when PGA is the substrate. Figure 3D. Comparison of half-life (t_1/2_) with temperature between APL and NP-APL. Figure 3E. Comparison of half-life (t_1/2_) with temperature between WT and NP-WT.

For NP-APL, activity increased by 33% at 90°C as compared to 50°C. In contrast the activity of untreated APL (assayed in the presence of 1 mM CaCl_2_) decreased by 59.8% at 90°C as compared to 50°C. Similarly for NP-WT, activity increased by 26% at 90°C as compared to 50°C. The loss of activity of untreated WT (assayed in the presence of 1 mM CaCl_2_) was 60% (Figure 3A).It is clear from these data that NP had a significant role in conferring thermostability to both APL and WT. The extent of NP induced activation was similar in both WT and APL. However, CaCl_2_ was ineffective in maintaining enzyme activity at all temperatures in the range of 50–90°C. Our findings are in keeping with those of [19] for the PL from *Bacillus pumilus*. This suggests that irrespective of the source of the enzyme PL the co-factor (Ca) in the nano form could confer stability to the enzyme at elevated temperatures. Pectate lyase A (Pel A) of *Aspergillus nidulans* exhibited its optimum level of activity over the range of pH 7.5–10 at 50°C [26]. Pectic acid lyase (PAL) isolated from three isolates of *Syncephalastrum racemosum* showed optimum activity at 30°C [27]. Thus both the NP-APL and NP-WT are distinctive in exhibiting high activity at elevated temperatures of upto 90°C. The use of the co-factor Ca as a NP thus confers this unique property to the *Macrophomina* PL, which is being reported by us for the first time.

### Retention of Activity at 90°C by NP-PL (PGA as Substrate)

With PGA as substrate NP-APL retained 100% of its activity at the end of two hours (incubation at 90°C) the corresponding values for this system at the end of 4 hours and 6 hours of incubation were 95.4% and 95.2% respectively. (Fig: 3C) Thus over a total period of 6 hours the drop in enzyme activity was only about 5%. Similarly for the NP-WT system a 100% retention of activity was observed at end of 2 hours of incubation at 90°C with corresponding values of 100.8% and 98.7% retention in activity at the end of 4 hours and 6 hours of incubation. Thus a loss of only 2% activity was seen at the end of 6 hours.(Fig: 3C). Since the activity values at the start of the experiment were about 80 units/ml for NP-APL and NP-WT, it is evident that the high activity conferred by NP persisted over a fairly long time interval. On the other hand for the untreated WT and APL there was a drop in activity of 57.3% and 54.7% respectively under the conditions as above. Since both WT and APL showed greatly diminished activity (to the extent of 50% loss) after the initial exposure to 90°C the final activity of these two systems was very feeble. Thus, the NP-PL system conferred stability at high temperature to both the WT and the (functionally impaired) APL. The NP-APL and NP-WT systems showed a similar trend when pectin was used as a natural substrate. (Fig: 3B) This finding could be utilized for applications involving reactions for prolonged periods at high temperature.

### Kinetics and Activation Energy NP-WT and NP-APL

#### K_m_, V_max_ and activation energy (E_a_)

For the NP-WT, the K_m_ value was 0.463 mg/ml at 50°C with a V_max_ value of 77.14 Units/ml. For the untreated WT, at 50°C the K_m_ and the V_max_ values were 0.549 mg/ml and 36.92 units/ml respectively. At 90°C, for the NP-WT, the K_m_ value was 0.184 mg/ml with a V_max_ value of 263 Units/ml. On comparing these systems, we observed that there was a 3.4 fold increase in V_max_ with 60.25% decrease in K_m_ for the NP-WT at 90°C than the untreated WT at 50°C. In contrast to this, the untreated WT had 40.07% decreased V_max_ and 34.79% increased K_m_ at 90°C compared to the untreated WT at 50°C. (Table 4).

**Table 4:** Km-Vmax and the Activation energy values of the PL and NP-PL systems of the attenuated and the WT fungus.

Enzyme System	K_m_ @ 50°C(mg/ml)	V_max_ @50°C (unit/ml)	K_m_ @90°C(mg/ml)	V_max_ @90°C(unit/ml)	E_a_ (KJmol^−1^)
**APL**	1.25	16.66	3.70	8.10	18.25
**NP-APL**	0.47	40.02	0.31	214	−17.25
**WT**	0.55	36.92	0.74	21.9	14.32
**NP-WT**	0.46	77.14	0.184	263	−25.14

For the NP-APL, the K_m_ value was 0.471 mg/ml at 50°C with a V_max_ value of 40.02 Units/ml. For the untreated APL,at 50°C the K_m_ and the V_max_ values were 1.25 mg/ml and 16.66 units/ml respectively. At 90°C, for the NP-APL, the K_m_ value was 0.31 mg/ml with a V_max_ value of 214 Units/ml. On comparing these systems, we observed that there was a 12.8 fold increase in V_max_ with 75.2% decrease in K_m_ for the NP-APL at 90°C than the untreated APL at 50°C. In contrast to this, the untreated APL had 51.33% decreased V_max_ and 3 fold increase in K_m_ at 90°C compared to the untreated APL at 50°C.(Table 4).

The NP-APL and the NP-WT systems had similar patterns of increased V_max_ and lowered K_m_ at 90°C than their corresponding untreated sets suggesting that NP could activate the enzymes at the higher temperatures by increasing the affinity for the substrate while simultaneously increasing its conversion rate.

Thus at temperatures which normally favours denaturation of the enzyme system with loss of activity, the incorporation of NP’s stabilizes the enzyme. It was also significant that at the normal assay temperature (50°C) APL had almost double the K_m_ value for the WT with almost half the V_max_ values. The functional impairment of the APL was thus an outcome of a combination of diminished substrate affinity and conversion kinetics. This aspect could possibly be linked to the structural changes observed and detailed in a later section.

The activation energy (E_a_) of NP-APL was found to be almost 35 fold less than that of untreated APL. From the Arrhenius plot,The APL showed an (E_a_) value of 18.245 kJ/mole whereas NP-APL showed an (E_a_) value of −17.251 kJ/mole. Again, from the Arrhenius plot, the (E_a_) value of the WT was found out to be 14.327 kJ/mole whereas in case of the NP-WT, (E_a_) value was found out to be −25.143 kJ/mole which was 39 fold less than that of the untreated WT.(Table 4).

#### Activation/Inactivation kinetics of PL and NP-PL systems

**Figure 4A:**
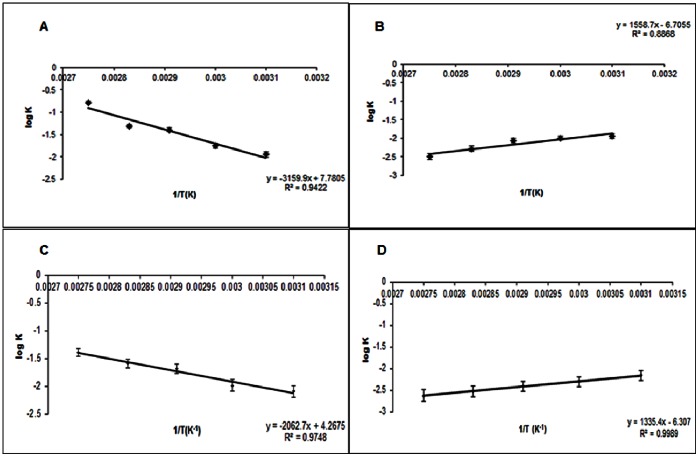
Arrhenius plot for deactivation energy(E_d_) for APL. Figure 4B. Arrhenius plot for deactivation energy(E_d_) for NP-APL. Figure 4C. Arrhenius plot for deactivation energy(E_d_) for WT. Figure 4D. Arrhenius plot for deactivation energy(E_d_) for NP-WT.

The kinetics of NP-WT and NP-APL systems were examined within the temperature range between (50–90) °C. Semi-logarithmic plots of residual activity versus time between (50–90) °C for all the systems viz.(APL,NP-APL,WT and NP-WT systems) were found to be linear (Fig. 4A and Fig. 4B; Fig. 4C and Fig. 4D) The plots suggested that APL and WT were heat inactivated with first order kinetics, whereas the NP-APL and NP-WT were heat activated with first order kinetics. The half-life (t_1/2_) values were calculated according to the plots (Table 5 and 6, Table 7 and 8).It was observed, that (t_1/2_) values increased with increasing temperature for the NP-APL and NP-WT. (Fig: 4A and 4B).

**Table 5:** Variation of kinetic parameters within (50–90) °C for NP-APL.

Pre-Incubation Temperature(K)	Dissociation Constant[k] (min^−1^)	Half Life [t_1/2_] (min)	E_d_ (kJ/mol)	ΔH (kJ/mol)	ΔG (kJ/mol)	ΔS (J/mol/k)
323	0.01124±0.0031	61.65±1.2	29.84±2.49	27.15±1.21	91.38±0.51	−198.83±2.13
333	0.01012±0.0030	68.47±1.5		27.06±1.22	94.57±0.34	−202.74±3.006
343	0.0086±0.0023	80.58±1.3		26.99±1.21	98.01±0.51	−207.05±2.86
353	0.0052±0.0027	133.2±0.9		26.91±1.21	102.42±0.51	−213.92±2.74
363	0.0032±0.0017	216.6±2.2		26.82±1.11	106.83±0.43	−220.39±4.11

**Table 6:** Variation of kinetic parameters within (50–90) °C for NP-WT.

Pre-Incubation Temperature(K)	Dissociation Constant[k] (min^−1^)	Half Life [t_1/2_] (min)	E_d_ (kJ/mol)	ΔH (kJ/mol)	ΔG (kJ/mol)	ΔS (J/mol/k)
323	0.006829±0.0013	101.5±0.9	25.56±1.33	22.88±0.38	92.69±0.75	−216.13±2.13
333	0.004959±0.03	139.7±1.0		22.80±1.36	96.53±0.92	−221.41±1.93
343	0.003852±0.01	179.8±1.0		22.72±1.73	100.24±0.34	−225.99±2.02
353	0.00291±0.09	238.5±1.3		22.64±0.37	104.06±0.84	−230.67±1.97
363	0.002340±0.02	296.1±0.8		22.55±2.13	107.74±0.84	−234.68±3.64

**Table 7:** Variation of kinetic parameters within (50–90) °C for APL.

Pre-Incubation Temperature(K)	Dissociation Constant[k] (min^−1^)	Half Life [t_1/2_] (min)	E_d_ (kJ/mol)	ΔH (kJ/mol)	ΔG (kJ/mol)	ΔS (J/mol/k)
323	0.01127±0.0002	61.49±1.3	−60.50±2.97	−63.19±1.27	91.38±0.59	−478.53±1.37
333	0.01757±0.0004	39.44±1.3		−63.27±2.28	93.05±0.27	−469.43±0.87
343	0.03982±0.0051	17.40±3.1		−63.35±3.23	98.99±0.54	−473.30±1.37
353	0.04846±0.0037	14.30±1.2		−63.44±2.19	95.84±1.28	−451.19±0.54
363	0.1641±0.0096	4.22±2.11		−63.52±1.87	94.96±1.54	−436.59±0.54

**Table 8:** Variation of kinetic parameters within (50–90) °C for WT.

Pre-Incubation Temperature(K)	Dissociation Constant[k] (min^−1^)	Half Life [t_1/2_] (min)	E_d_ (kJ/mol)	ΔH (kJ/mol)	ΔG (kJ/mol)	ΔS (J/mol/k)
323	0.00802±0.0043	86.40±1.6	−39.48±1.64	−42.17±2.87	92.27±1.24	−416.19±1.43
333	0.01030±0.0103	67.28±2.3		−42.25±1.48	94.51±1.43	−411.94±2.34
343	0.02053±0.0051	33.75±2.1		−42.33±3.15	95.46±3.27	−401.73±1.94
353	0.02545±0.0049	27.22±1.9		−42.42±2.67	97.70±1.27	−396.92±2.02
363	0.04043±0.014	17.14±2.4		−42.49±1.95	99.15±2.23	−390.20±2.64

The activity of APL was seen to decrease with increasing temperature as reflected in the half -life (t_1/2_) values. Beyond 60°C, enzyme activity was severely inhibited. On the contrary the thermal stability of NP-APL was found to increase with temperature. The deactivation energy (E_d_) of NP-APL was calculated from a linear part of Arrhenius plot and it was around 29.84 kJ/mol and (E_d_) of untreated enzyme was found to be −60.50 kJ/mol. (Figure: 4B) The half-life of enzyme inactivation was 61.6 min at 50°C for NP-APL as compared to the half- life of inactivation of 216.5 min at 90°C for the NP-APL. APL had a t_1/2 _at 90°C of 4.22 min which was roughly 51-fold lower than that of NP-APL which had a t_1/2_ of 216.56 min at 90°C.For the WT, (E_d_) of untreated enzyme was −39.481 kJ/mol whereas, for the NP-WT, the (E_d_) was calculated to be 25.569 kJ/mol (Table 6 and 8). The results suggest that NP confers stability (in terms of energy required to deactivate) to both WT and APL to a similar extent. This finding is remarkable as APL has *per se* about 30% lower (E_d_) as compared to WT. Half -life studies of the WT w.r.t NP-WT had a similar pattern to it. At 90°C, The NP-WT had a half-life of 296.07 min compared to a half-life of 17.14 min of the untreated WT.

### Entropy-Enthalpy Map

The entropy enthalpy map summarizes the thermodynamic changes associated with the enzymatic process in the presence and absence of the nanoparticle. In presence of NP, a compensatory behavior was evident. In absence of NP, a critical change in the entropy-enthalpy map was observed. Incidentally, the critical behavior corresponded to the onset of inactivation of the enzyme.

The entropy–enthalpy profile for the thermal inactivation process of NP-APL and NP-WT showed that, till 90°C there was entropy–enthalpy compensation. For the NP treated enzyme the ΔH and ΔS had opposite sign(s), implying a significant entropy enthalpy compensation [28], [29]_._ The profile remained monotonic in the temperature range 50°C to 90°C.

**Figure 5A:**
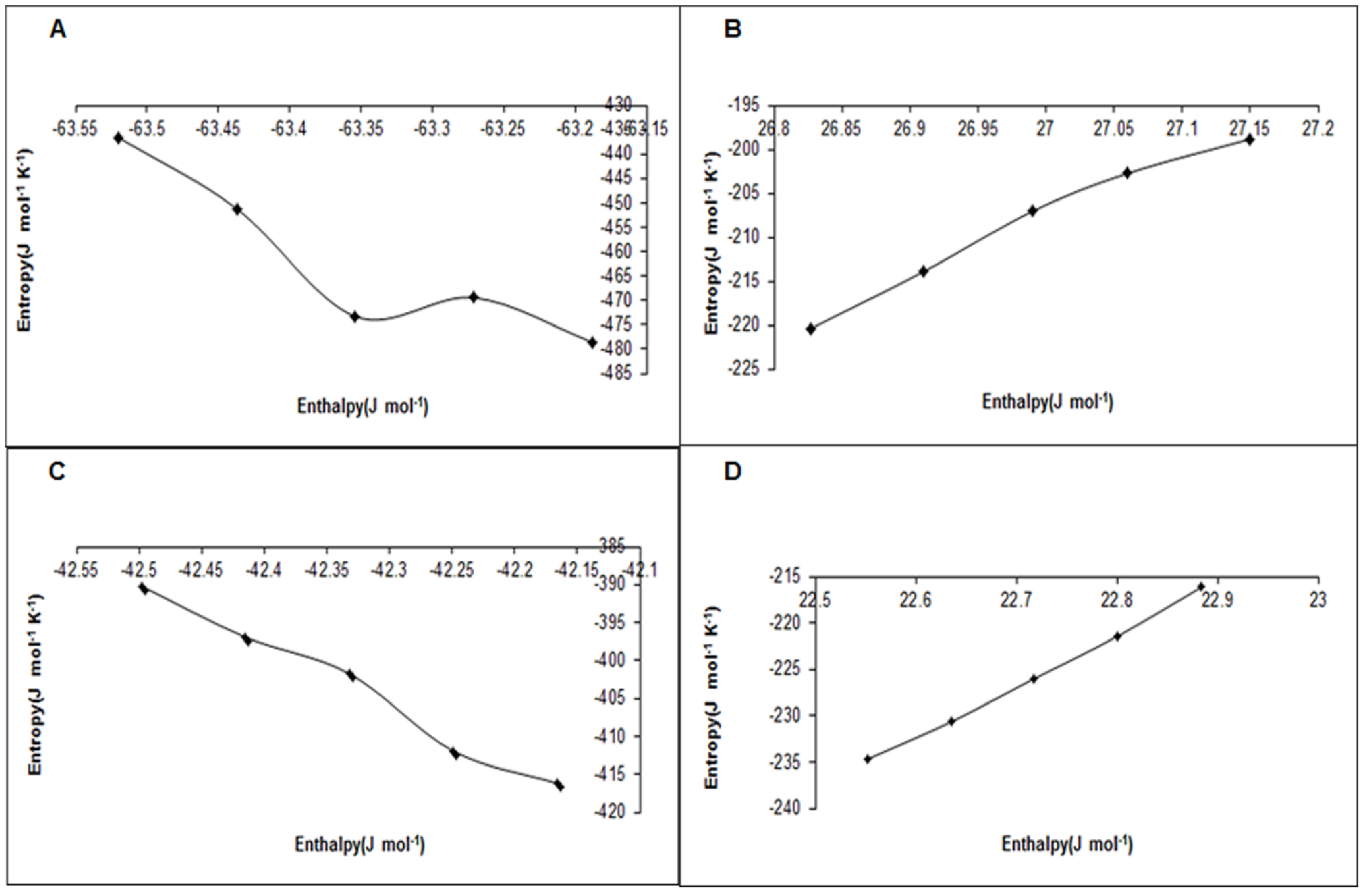
Entropy-Enthalpy compensation for APL. Figure 5B. Entropy-Enthalpy compensation for NP-APL. Figure 5C. Entropy-Enthalpy compensation for WT. Figure 5D. Entropy-Enthalpy compensation for NP-WT.

In contrary to this in case of APL and WT below 50°C, entropy-enthalpy compensation was operative (Fig. 5(a)–(d)). Beyond such critical point, the compensatory profile was lost, the entropic and enthalpic contributions assumed the same sign (both negative). The unfavorable entropic contribution perhaps indicated a loss of structure (and enzymatic activity) as maintenance of native structure normally is entropy driven process.

Identical signs of ΔH and ΔS generally imply a weak form of compensation [28], [29] where the free energy balance (negative free energy change) is obtained by loss of structure and activity. Thus, above 50°C, thermal stability of the untreated enzyme decreased rapidly due to the favorable situation that were generated for protein unfolding by the change of entropy and enthalpy of the reactions.

### Analysis of CD Structures of WT and APL as Influenced by NP and Temperature

From the circular dichroism (CD) data (Table 9), for APL (at 50°C) the (β) strand content (per 100 residues) was about 5 fold less compared to the WT. However, for NP-WT and NP-APL these values became almost similar for the two systems.

**Table 9:** Analysis of CD structures of the PL and NP-PL systems.

Enzyme System	(β) strand content (per 100 residues) at 90°C(-NP)	(β) strand content (per 100 residues) at 90°C(+NP)	(β) strand content (per 100 residues) at 50°C(-NP)	(β) strand content (per 100 residues) at 50°C(+NP)
**WT**	1.26	8.44	3.94	4.18
**APL**	0.11	8.47	0.66	3.35

Since, pectate lyase is a protein stabilized by beta (β) strands (Source: RCSB Protein Data Bank; URL:www.rcsb.org/pdb/explore/derivedData.do?structureId=1NHC; Domain Annotation : SCOP,Classification (Version 1.75) Accessed 2012 Nov, this would explain the basis for the functional impairment of APL and the recovery of activity affected by NP. At 90°C both the WT and the APL show loss of (β) strand content by about 33% and 16% respectively. This correlates to the loss of activity of WT and APL as shown (Fig: 1B). However at 90°C the strand content was not only similar for NP-WT and NP-APL but exceeded (by a factor of about 2) the values obtained for the NP-WT/NP-APL at 50°C. This probably was a factor contributing to the enhanced activity and thermostability of NP-PL at the higher temperature. However, the reasons for the increase in (β) strand content could not be understood.

## Discussion

Upon alignment of the protein sequences of the WT and APL, a number of amino acid deletions and substitutions were found in the APL with respect to the WT. Most of the deletions were found to be at the alanine and arginine positions. But since the mutation is completely random, it is difficult to make a valid conjecture for the deletions at this moment.

The optimum activity of purified APL was obtained at a NP concentration of 10.5 µg/ml. This increase in activity of the NP-APL was 19 fold higher as compared to the untreated APL at 50°C. This trend was also followed for WT where the increase was about 9 fold (possibly because of the higher base effect).

With PGA as substrate NP-APL and NP-WT retained 100% of their activity at the end of two hours of incubation at 90°C. After 4 hours and 6 hours of incubation, there was a loss of activity by 2%–5% (appx) for both NP-APL and NP-WT. On the other hand for the untreated WT and APL there was a drop in activity of 57.3% and 54.7% respectively under these conditions. The stability conferred to both APL and WT by NP at 90°C can thus be utilized for conditions involving high temperatures for prolonged periods. These features are supported by the biochemical parameters that were examined. The lowering of the activation energy (E_a_) values of NP-APL and NP-WT, their low K_m_ and high V_max_ values signify that nanoparticle conjugated PL systems would be more easily activated than the untreated one, irrespective of whether the enzyme was sourced from WT or APL.

The most significant aspect of these findings is the restoration of function to the functionally impaired APL to bring it to comparable levels with the WT. This indicates a role for the nano-particle which may be extended to other functionally impaired proteins.

The gradual decrease of dissociation constant (k) value with increasing temperature, the high deactivation energy (E_d_) and the increase of t_1/2_ value with temperature of NP-APL and NP-WT suggests a model where NP stabilizes the enzyme complex at higher temperatures, thereby requiring higher energy to deactivate it. In comparison, the APL system needed very low energy to deactivate and the low t_1/2_ value at higher temperature indicates a more rapid loss of function.

From the entropy-enthalpy map, it was evident that in presence of NP, that ΔH-ΔS exhibited a compensatory relationship as the linearity of the ΔH-ΔS plot was maintained at a higher temperature. In absence of NP, a critical change in the entropy-enthalpy map was observed. The unfavourable entropic contribution perhaps indicated a loss of structure (and enzymatic activity) as maintenance of native structure normally is an entropy-driven process.

Our studies show that a functionally impaired pectate lyase (APL) could regain activity in the presence of NP. Such activity was retained at a relatively high temperature of 90°C and was comparable to the activity of the WT. This phenomenon was linked to the enhanced formation of beta (β) strands in the impaired enzyme at both moderate (50°C) and high (90°C) temperatures. This is to our knowledge, the first report of a nano-particle mediated activity enhancement and thermostability for an enzyme system intrinsically deficient in activity Though earlier reports [19], [30] from our laboratory suggested that nano-particles were capable of enhancing the activity and thermal stability of pectate lyase and laccase these related to the wild type enzymes. However, the restorative action of nano-Ca on APL was specific for the enzyme system (nano Cu had no effect; data not given) thereby opening a field for further work of this type.
